# Editorial: Role of probiotics and probiotics' metabolites in food and intestine

**DOI:** 10.3389/fmicb.2023.1183550

**Published:** 2023-03-24

**Authors:** Vincenzina Fusco, Rina Wu, Wenyi Zhang, Qixiao Zhai

**Affiliations:** ^1^Institute of Sciences of Food Production of the National Research Council of Italy (CNR-ISPA), Bari, Italy; ^2^Shenyang Agricultural University, Shenyang, China; ^3^Key Laboratory of Dairy Biotechnology and Engineering, Ministry of Education, Hohhot, China; ^4^Key Laboratory of Dairy Products Processing, Ministry of Agriculture and Rural Affairs, Inner Mongolia Agricultural University, Hohhot, China; ^5^Jiangnan University, Wuxi, China

**Keywords:** probiotics, prebiotics, food, intestine, metabolites

The emergence and exploration of new disciplines and technologies have provided new ideas and opportunities for probiotic science and industry development. The interaction between diet and intestinal flora has become a new target for human health regulation. Recently, probiotic supplements have received increasing attention as an important tool to modulate the gut microbiota (Brito Sampaio et al., 2022). Lactic acid bacteria (LAB) are powerful probiotics in the intestinal tract that can participate in metabolic regulation by directly or indirectly influencing the inhibition or activation of the signaling pathways. LAB can synthesize a variety of active metabolites, producing short-chain fatty acids, vitamins, enzymes, organic acids and antibacterial peptides. These metabolites can regulate the intestinal epithelium's barrier function and provide health benefits to the host.

Studies have found that probiotics have a variety of biological activities (Brito Sampaio et al., 2022; Leite de Souza et al., 2022). However, their application is still limited due to the poor colonization and the unclear mechanism of the induction of metabolites in the host involved in the interaction between pathogens and bacterial communities, etc. Therefore, this Research Topic aimed to collect new studies focused on probiotics and probiotics' metabolites in food and intestine with combined phenotyping, genotyping and targeting strategies as well as the multi-omics technologies.

Chen X. et al. assessed the effects of alkali stress on the growth and menaquinone-7 metabolism of *Bacillus subtilis natto*, whereas He et al. investigated the effects of *Akkermansia muciniphila* on gut microbiota and disease-related biomarkers in murine model, finding that this microorganism alters gut microbiota and immune system to improve cardiovascular diseases.

Li et al. demonstrated that autoinducer-2 exporters (AI-2E) family transporter protein in *Lactobacillus acidophilus* exhibits AI-2 exporter activity and relate with intestinal juice resistance of the strain.

Hu P. et al. isolated and identified *Rhodotorula mucilaginosa* TZR2014 and assessed its function as well as its effects on the growth and helath of weaned piglets.

Ke et al. demonstrated the inhibition of *Cronobacter sakazakii* in an infant simulator of the human intestinal microbial ecosystem using a potential synbiotic consisting of six lactic acid bacteria (LAB) strains and Vivinal GOS.

Hu Y. et al. demonstrated the alleviating effects of Selenium-enriched *Bifidobacterium longum* DD98 on dextran sulfate sodium-induced colitis in mice and explored the underlying mechanism.

Zhao et al. investigated the alleviating effects of gut micro-ecologically regulatory treatments on onstipation in mice.

Chen P. et al. investigated the regulations of lipid metabolism of broilers by *Bacillus amyloliquefaciens* TL both *in vivo* and *in vitro*, whereas Wang et al. demonstrated the exertion by *Lactobacillus mucosae* of different antiviral effects on respiratory syncytial virus infection in mice.

Sheng et al. discovered a novel exopolysaccharide derived from the probiotic *Lactobacillus pantheris* TCP102 strain exerting immune-enhancing and anticancer activities.

Finally, Liu et al. provided a review on antifungal mechanisms and application of Lactic Acid Bacteria in bakery products, whereas Shi et al., provided a review on the recent advances in the roles of microorganisms in fermented foods based on multi-omics data.

## Author contributions

VF conceived and wrote the manuscript. All authors revised the manuscript. All authors contributed to the article and approved the submitted version.
